# Association between the triglyceride–glucose index and the risk of acute kidney injury in critically ill patients with acute pancreatitis: a retrospective study

**DOI:** 10.1186/s40560-025-00779-x

**Published:** 2025-02-05

**Authors:** Zheng Wang, Haoyu Zhang, Xiaozhou Xie, Jie Li, Yuchen Jia, Jiongdi Lu, Chongchong Gao, Feng Cao, Fei Li

**Affiliations:** 1 Department of General Surgery, Xuanwu Hospital, Capital Medical University, No.45 Changchun Street, Xicheng District, Beijing, 100053 China; 2 Clinical Center for Acute Pancreatitis, Capital Medical University, Beijing, China

**Keywords:** Triglyceride–glucose index, Acute pancreatitis, Acute kidney injury, MIMIC-IV, Critical illness

## Abstract

**Background:**

The triglyceride–glucose (TyG) index is increasingly recognized for its ability to predict cardiovascular and metabolic risks. This study investigated the correlation between the TyG index and the risk of acute kidney injury(AKI) in critical ill patients with acute pancreatitis(AP).

**Methods:**

The Medical Information Mart for Intensive Care IV database was retrospectively searched to identify AP patients hospitalized in the intensive care unit. The primary outcome measure was the incidence of AKI. The secondary endpoint was in-hospital mortality and the rate of renal replacement therapy(RRT) use. Cox regression analysis and restricted cubic spline were used to analyze TyG index association with AKI risk. Kaplan–Meier survival analysis was performed to assess the incidence of endpoints in the different groups.

**Results:**

A total of 848 patients were enrolled. The incidence of AKI was 61.56%.The in-hospital mortality was 11.69%. Kaplan–Meier analysis showed that the TyG index ≥ 8.78 group has a high incidence of AKI and high risk of requiring RRT (*P* < 0.001). Multivariable Cox regression analysis showed whether TyG index was a continuous variable (HR, 1.65 [95% CI 1.10–2.48], *P* = 0.015) or a categorical variable (HR, 1.72 [95% CI 1.09–2.79], *P* = 0.028), and the TyG index was independently associated with the risk of AKI in AP patients. The restricted cubic splines model illustrated the linear relationship between higher TyG index and increased risk of AKI in this specific patient population.

**Conclusions:**

High TyG index is an independent risk factor for AKI in critical ill patients with AP. Assessing the TyG index may be beneficial for early stratification and interventions to improve prognosis.

## Background

Acute pancreatitis(AP) is one of the leading causes of hospitalization for gastrointestinal diseases, accounting for approximately 300 000 emergency department visits in the United States each year [1]. Most AP cases are mild and self-limiting, requiring only brief hospitalization. Severe pancreatitis occurs in about 15–20% of patients and is associated with multiorgan failure and poor prognosis [2]. Acute kidney injury (AKI) is a common and significant complication of AP, which is caused by intra-abdominal hypertension or abdominal compartment syndrome [3]. It is estimated that about 7% to more than 20% of hospitalized patients with AP develop AKI [4, 5]. This complication occurs in nearly 70% of AP cases admitted to the intensive care unit(ICU), the occurrence of AKI significantly increases the risk of death in AP patients [6]. Therefore, identifying AP patients at high risk of developing AKI in the ICU is essential to improve their prognosis.

Insulin resistance(IR) and dyslipidemia have already been proven essential in the development and progression of kidney disease [7, 8]. The triglyceride–glucose (TyG) index is being extensively investigated as a biomarker for identifying IR [9, 10]. The TyG index was calculated using the formula ln [fasting triglyceride (mg/dl) x fasting blood glucose (mg/dl)/2]. Previous studies have shown that TyG index is associated with the incidence of chronic kidney disease and acute kidney injury [11, 12]. Furthermore, the TyG index is associated with poor prognosis in patients with AP, and it is an effective biomarker for identifying severe AP [13, 14]. But the relationship between the TyG index and the risk of AKI in critically ill patients with AP remains unclear. Therefore, we undertook a retrospective cohort study aiming to investigate the prognostic value of the TyG index in the development of AKI in critically ill patients with AP.

## Materials and methods

### Data source

All data for this study were obtained from and the Medical Information Mart for Intensive Care IV (MIMIC-IV) database, a collaborative project between the Massachusetts Institute of Technology and Beth Israel Deaconess Medical Center. This database includes information on all patients admitted to the Beth Israel Deaconess Medical Center during the years from 2008 to 2019. Data for this study were obtained from publicly available sources with de-identified information, thus exempting ethical approval and informed consent requirements. Access to this platform is granted upon completion of the online course provided by the National Institutes of Health. The first author of the present study, Zheng Wang, successfully passed the database usage examination and obtained a license for use of the MIMIC-IV database (User ID: 12847014).

### Study population

**Fig. 1:**
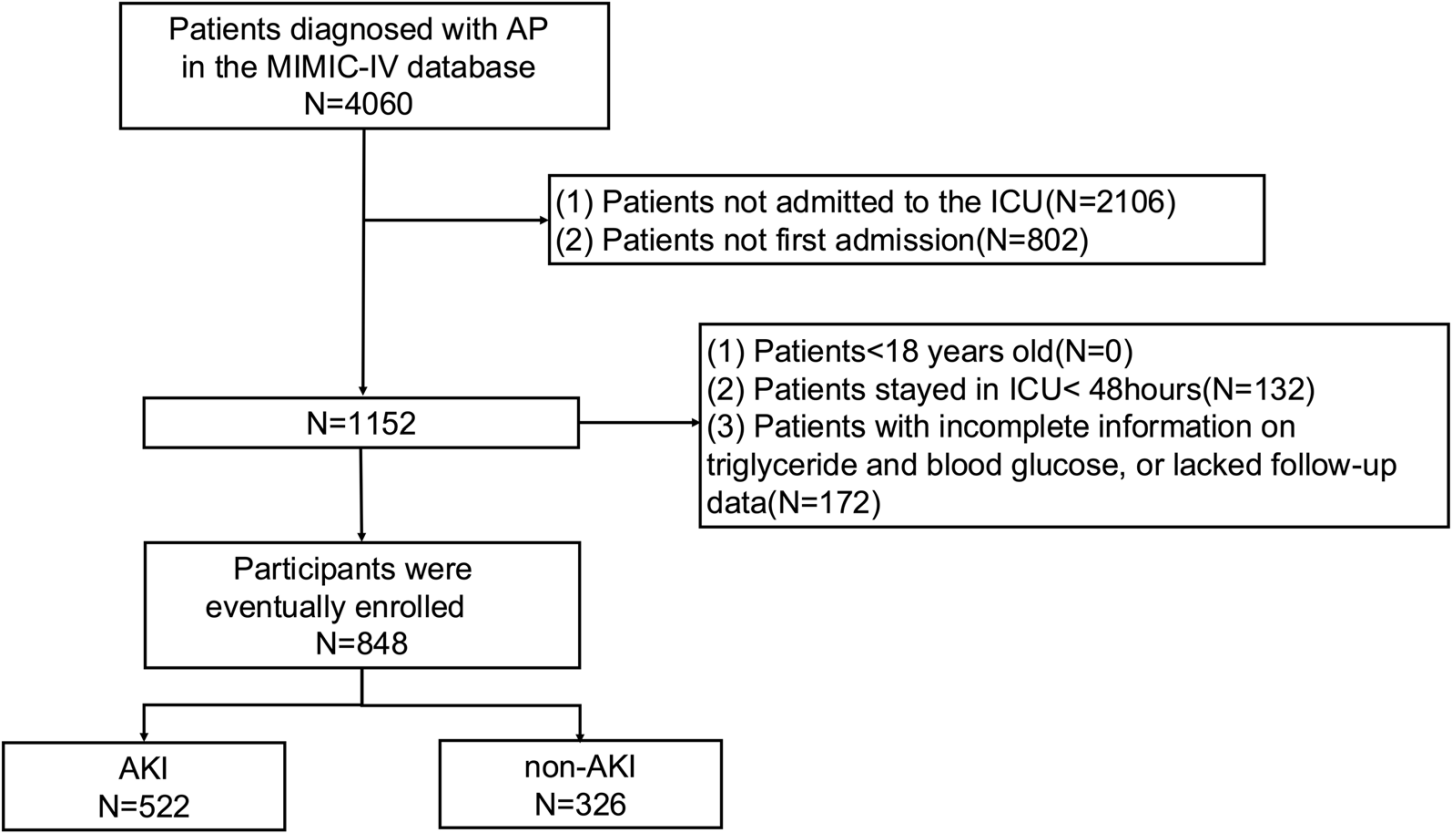
Flow chart of patient selection. *AP* acute pancreatitis, *MIMIC-IV* Medical Information Mart for Intensive Care IV, *ICU* intensive care unit, *AKI* acute kidney injury

The MIMIC-IV database was retrospectively searched to identify patients hospitalized in the ICU. A total of 4060 patients diagnosed with AP were extracted according to the International Classification of Diseases versions 9 and 10. AP was diagnosed based on the presence of two of the following three features: (1) typical abdominal pain, (2) serum amylase and/or lipase≥3 times the upper normal limit, and (3) radiologic findings. Patients were excluded if they were younger than 18 years of age at initial admission, had an ICU stay of less than 48 h, had incomplete information on triglyceride (TG) and blood glucose, or lacked follow-up data. Patients with multiple ICU admissions for AP, where only data from the first admission were used. Ultimately, 848 patients were included in the study (Fig. 1).

### Variable extraction

All variables were extracted from the MIMIC-IV database using Navicat Premium software (version 16) and PostgresSQL software (version 13.7.2) through the execution of a Structured Query Language. The data extracted included various demographic characteristics including; sex, age, Body mass index (BMI). Laboratory variables within the first 24 h of ICU admission including; hemoglobin, red blood cell (RBC), white blood cell (WBC), neutrophils, hemoglobin, lymphocytes, platelet count, glucose, high density lipoprotein cholesterol (HDL), low density lipoprotein cholesterol (LDL), albumin, total cholesterol (TC), TG, total bilirubin, creatinine, potassium, blood–urea–nitrogen (BUN), aspartate aminotransferase (AST), alanine aminotransferase (ALT), alkaline phosphatase (ALP), c-reactive protein (CRP). Severity on admission including; simplified acute physiology score II (SAPSII), systemic inflammatory response syndrome (SIRS) score, acute physiology score III (APSIII) and sequential organ failure assessment (SOFA) score. Comorbidities including; acute kidney injury, atrial fibrillation, hypertension, diabetes, myocardial infarction, chronic obstructive pulmonary disease (COPD), chronic kidney disease (CKD), and the appliaction of renal replacement therapy(RRT). Given the notable prevalence of missing values for laboratory indicators in the MIMIC-IV database, the percentage of missing values for each continuous variable was computed. To address potential bias arising from sample exclusion, variables with more than 20% missing values were excluded. Variables with less than 20% missing data underwent multiple imputation using the random forest algorithm.

### Primary endpoints and secondary endpoints

The primary outcome was the incidence of AKI. AKI is defined by Kidney Disease: Improving Global Outcomes guideline criteria. AKI is defined as an increase in serum creatinine level of ≥ 0.3 mg/dl within 48 h; or ≥ 1.5 times the baseline within 7 days; or urine volume of < 0.5 ml/kg/h for 6 h or more[15]. The secondary outcome encompassed in-hospital mortality for both the whole study population and the AKI subset and RRT application in AKI population.

### Statistical analysis

For baseline characterization, continuous variables were expressed as mean ± standard deviation (SD) for normally distributed variables, median (interquartile range, IQR) for non-normally distributed continuous variables, and numbers (%) for categorical variables. The *T* test or one-way ANOVA was used to compare continuous variables, while Pearson’s *χ*^2^ test was used to compare categorical variables when examining baseline characteristics. The Kaplan–Meier survival analysis was employed to estimate the incidence of AKI, the in-hospital mortality among whole study population and AKI population based on the TyG index, and the use of RRT among AKI population. Cox proportional hazards models were utilized to compute the hazard ratio (HR) and the 95% confidence interval (CI) for the TyG index and incidence of AKI within each subgroup. Crude models did not adjust for any potential confounders. Model 1 adjusted for age, sex, and BMI. Model 2 was further adjusted for laboratory variables (hemoglobin, RBC, WBC, neutrophils, lymphocyte, HDL, LDL, TC, TG, total bilirubin, creatinine, potassium, BUN, AST, ALT, ALP, CRP) and comorbidities(hypertension, atrial fibrillation, myocardial Infarction, COPD, CKD, diabetes). Restricted cubic spline (RCS) was also used to further validate the potential nonlinear relationships between TyG index levels and the incidence of AKI. To ensure the robustness of our findings, we performed stratified analyses based on several factors, including age, gender, BMI, hypertension, diabetes and CKD. Data analysis was performed using R software version 4.1.2 (R-project; R foundation for Statistical Computing, Vienna, Austria) and X-tile version 3.6.1. A two-sided *P* value of < 0.05 was considered statistically significant.

## Results

### Baseline characteristics

A total of 4060 patients diagnosed with pancreatitis were extracted from the database. According to the inclusion and exclusion criteria, 848 patients were included in the study, and the patient screening flow chart is shown in Fig. 1. The average age was 56.89 ± 16.76 years, and 497 (58.61%) were male. The median TyG index was 9.02 (IQR, 8.53, 9.64). The incidence of AKI was 61.56%.The in-hospital mortality was 11.69%.

The X-tile software was utilized to establish the optimal TyG cutoff value. To ensure the accuracy of the results, we randomly divided the original data into a training set (70%) and a test set (30%). The X-tile software was used to determine the best cutoff point (TyG index = 8.78) in the training set(log-rank *P* < 0.001), and its discrimination effect was verified in the test set. In the test set, the incidence of AKI in the high TyG group (TyG index ≥ 8.78) and the normal TyG group (TyG index < 8.78) was 64.3% and 25.8%, respectively. The chi-square test showed a P value of less than 0.001, indicating that the cutoff point was also statistically significant in the independent data. K-fold cross validation (*K* = 5) was used to evaluate the robustness of the optimal cutoff point. After each folding training, the optimal cutoff point was recalculated and compared with the cutoff point of the overall data (8.78). The results show that the cutoff point in different folds varies between 8.75 and 8.81 with small deviations, which supports the robustness of the 8.78 cutoff point.

The enrolled patients into two groups according to the: the normal TyG group (TyG index < 8.78) and the high TyG group (TyG index ≥ 8.78). Table 1 presents the baseline characteristics of the patients grouped according to the the TyG index. Compared with TyG index < 8.78 group, the TyG index ≥ 8.78 group were generally younger, had a higher BMI, APSIII, SIRS scores and a higher prevalence of diabetes. In terms of laboratory parameters, WBC, TG, TC, creatinine, BUN, glucose, and LDL in the TyG index ≥ 8.78 group were higher than those in the TyG index < 8.78 group, while total bilirubin, and HDL were on the contrary. The incidence of AKI in TyG index ≥ 8.78 group was significantly higher than that in TyG index < 8.78 group(65.46 vs 55.25%, *P* = 0.003).

**Table 1:** Baseline characteristics according to TyG index

	Overall, *N* = 848	TyG indx < 8.78, *N* = 324	TyG index ≥ 8.78, *N* = 524	*P* value
Age, years	56.89 ± 16.76	59.82 ± 18.47	55.50 ± 16.21	< 0.001
Sex(male)	497 (58.61%)	171 (52.78%)	326 (62.21%)	0.007
BMI	27.98 (24.13, 32.70)	27.13 (23.25, 31.56)	29.45 (25.29, 34.09)	< 0.001
RBC,m/uL	4.12 ± 0.82	4.12 ± 0.74	4.13 ± 0.87	0.905
WBC,K/uL	9.20 (6.60, 13.50)	7.80 (5.90, 11.80)	8.55 (6.33, 12.08)	< 0.001
Neutrophils,%	73.64 ± 14.83	73.32 ± 15.24	74.76 ± 15.02	0.600
Lymphocyte,%	17.21 ± 12.48	18.89 ± 13.02	16.19 ± 12.20	0.114
Hemoglobin,g/dL	12.55 ± 2.37	12.51 ± 2.23	12.58 ± 2.45	0.676
Platelets,(K/uL)	246.42 ± 123.64	253.21 ± 123.09	242.90 ± 122.23	0.178
TG,mg/dL	136.00 (90.00, 218.00)	85.00 (66.00, 105.00)	189 (140.00, 310.00)	< 0.001
TC,mg/dL	163.00 (128.00, 203.00)	153.00 (122.00, 186.00)	175.00 (135.00, 219.00)	< 0.001
Potassium, mmol/L	4.22 ± 0.79	4.17 ± 0.72	4.24 ± 0.82	0.189
Albumin,g/dL	3.55 ± 0.79	3.58 ± 0.78	3.53 ± 0.79	0.314
Total Bilirubin,mg/dL	23.60 ± 5.42	24.88 ± 4.91	22.90 ± 5.58	< 0.001
Creatinine,mg/dL	1.00 (0.70, 1.40)	0.90 (0.70, 1.20)	1.00 (0.80, 1.50)	< 0.001
ALT, U/L	32.25 (20.32, 75.80)	30.89 (18.91, 74.54)	33.87 (21.34, 78.09)	0.195
AST, U/L	42.21 (24.47, 103.98)	38.21 (24.36, 88.81)	46.02 (24.40, 107.82)	0.166
BUN, mg/dL	17.22 (12.87, 27.00)	16.62 (11.21, 23.80)	18.34 (12.18, 31.19)	0.003
Glucose,mg/dL	118.92 (96.35, 159.90)	100.52 (87.10, 118.89)	139.10 (107.23, 193.76)	< 0.001
HDL, mg/dL	47.25 ± 24.01	55.50 ± 25.91	41.76 ± 21.52	< 0.001
LDL, mg/dL	87.14 ± 36.27	82.92 ± 33.10	91.21 ± 38.33	0.004
ALP, U/L	91.26 (66.10, 144.50)	90.00 (66.28, 150.09)	91.10 (67.17, 143.25)	0.768
CRP, mg/L	18.19 (4.20, 79.03)	21.29 (4.10, 82.21)	16.21 (4.09, 68.27)	0.556
APSIII	47.00 (34.00, 66.00)	43.00 (33.00, 63.00)	49.00 (35.00, 67.00)	0.0037
SIRS	3.00 (2.00, 3.00)	3.00 (2.00, 3.00)	3.00 (2.00, 4.00)	0.005
SAPSIII	32.00 (23.00, 43.00)	31.00 (23.00, 41.00)	33.00 (22.00, 44.00)	0.705
SOFA	1.00 (0.00, 3.00)	1.00 (0.00, 3.00)	1.00 (0.00, 3.00)	0.145
Hypertension	217 (25.59%)	79 (24.38%)	138 (26.34%)	0.527
Atrial Fibrillation	174 (20.52%)	75 (23.15%)	99 (18.89%)	0.136
Myocardial Infarction	43 (5.07%)	11 (3.40%)	32 (6.11%)	0.080
COPD	88 (10.38%)	29 (8.95%)	59 (11.26%)	0.284
Diabetes	149 (17.59%)	54 (16.67%)	95 (18.13%)	0.241
RRT	103 (12.15%)	12 (3.70%)	91 (17.37%)	< 0.001
CKD	80 (9.43%)	27 (8.33%)	53 (10.11%)	0.389
AKI	522 (61.56%)	179 (55.25%)	343 (65.46%)	0.003
In-hospital mortality	99(11.69%)	24 (7.41%)	75 (14.31%)	0.002

Continuous variables were expressed as mean ± standard deviation (SD) for normally distributed variables, median (IQR) for non-normally distributed continuous variables, and numbers (%) for categorical variables. The *T* test or one-way ANOVA was used to compare continuous variables, while Pearson’s *χ*^2^ test was used to compare categorical variables when examining baseline characteristics

*BMI* body mass index, *RBC* red blood cell, *WBC* white blood cell, *TG* triglyceride, *TC* total cholesterol, *ALT* alanine aminotransferase, *AST* aspartate aminotransferase, *BUN* blood–urea–nitrogen, *HDL* high density lipoprotein cholesterol, *LDL* low density lipoprotein cholesterol, *ALP* alkaline phosphatase, *CRP* C-reactive protein, *APSIII* acute physiology score III, *SIRS* systemic inflammatory response syndrome score, *SAPSII* simplified acute physiology score II, *SOFA* sequential Organ Failure Assessment score, *COPD* Chronic obstructive pulmonary disease, *RRT* renal replacement therapy, *CKD* chronic kidney disease, *AKI* acute kidney injury

Table 2 lists the baseline characteristics of the AKI and non-AKI groups. The AKI group was older, had higher APSIII scores, SAPSIII scores, and SOFA scores, and higher incidence of CKD. In terms of laboratory indicators, AKI patients had higher levels of TG, serum potassium, creatinine, BUN and glucose, but low levels of RBC count, hemoglobin, platelets, total bilirubin, and HDL. The TyG index in AKI group was significantly higher than that in non-AKI group (8.81 [IQR 8.35, 9.42] vs. 9.11 [IQR 8.66, 9.80], *P* < 0.001).

**Table 2:** Baseline characteristics of the AKI and non-AKI groups

	Overall, *N* = 848	Non-AKI, *N* = 326	AKI, *N* = 522	*P* value
Age, years	56.89 ± 16.75	54.64 ± 17.41	58.32 ± 16.18	0.002
Sex(male)	497 (58.61%)	186 (57.06%)	311 (59.58%)	0.329
BMI	27.95 (24.12, 32.70)	27.88 (25.20, 31.53)	27.99 (23.77, 33.38)	0.876
RBC,m/uL	4.12 ± 0.82	4.20 ± 0.77	4.07 ± 0.85	0.024
WBC,K/uL	9.20 (6.60, 13.50)	9.20 (6.60, 13.50)	9.10 (6.60, 13.45)	0.785
Neutrophils,%	73.62 ± 14.83	74.23 ± 14.07	73.25 ± 15.29	0.341
Lymphocyte,%	16.86 ± 12.42	17.01 ± 12.48	16.77 ± 12.39	0.792
Hemoglobin,g/dL	12.55 ± 2.36	12.75 ± 2.26	12.42 ± 2.42	0.043
Platelets,(K/uL)	246.18 ± 122.56	261.39 ± 122.99	236.51 ± 121.43	0.004
TG,mg/dL	136.00 (90.00, 218.25)	115.00 (79.00, 188.00)	147.00 (98.00, 245.50)	< 0.001
TC,mg/dL	162.50 (128.00, 203.00)	168.50 (133.00, 203.00)	161.00 (124.00, 202.00)	0.629
Potassium, mmol/L	4.22 ± 0.78	4.15 ± 0.76	4.26 ± 0.79	0.048
Albumin,g/dL	3.55 ± 0.78	3.57 ± 0.78	3.53 ± 0.79	0.424
Total Bilirubin,mg/dL	23.59 ± 5.38	24.67 ± 4.79	22.90 ± 5.62	< 0.001
Creatinine,mg/dL	1.00 (0.70, 1.40)	0.80 (0.60, 1.00)	1.20 (0.80, 1.70)	< 0.001
ALT,U/L	32.00 (20.00, 75.00)	35.00 (19.50, 81.50)	30.00 (20.00, 73.50)	0.355
AST,U/L	42.00 (24.00, 103.00)	40.00 (25.00, 93.50)	43.00 (24.00, 105.75)	0.677
BUN,mg/dL	17.00 (12.00, 27.00)	14.00 (10.00, 20.00)	21.00 (14.00, 34.00)	< 0.001
Glucose,mg/dL	146.58 ± 99.63	137.73 ± 79.42	152.19 ± 110.25	0.027
HDL,mg/dL	46.66 ± 23.64	50.01 ± 23.83	44.96 ± 23.39	0.029
LDL,mg/dL	87.07 ± 36.10	89.72 ± 35.68	85.72 ± 36.30	0.264
ALP,U/L	91.00 (66.00, 144.00)	90.00 (66.00, 151.00)	91.00 (66.00, 140.50)	0.683
CRP,mg/L	18.45 (3.80, 79.05)	15.50 (2.20, 60.60)	19.60 (4.40, 80.50)	0.225
APSIII	47.00 (34.00, 66.00)	39.00 (31.00, 54.00)	51.00 (38.00, 75.00)	< 0.001
SIRS	2.77 ± 0.91	2.74 ± 0.88	2.79 ± 0.93	0.487
SAPSIII	32.00 (23.00, 43.00)	27.00 (20.00, 37.00)	36.00 (27.00, 46.00)	< 0.001
SOFA	1.00 (0.00, 3.00)	0.00 (0.00, 2.00)	2.00 (0.00, 4.00)	< 0.001
Hypertension	217 (25.59%)	88 (27.00%)	129 (24.71%)	0.538
Atrial Fibrillation	174 (20.52%)	57 (17.48%)	117 (22.41%)	0.067
Myocardial Infarction	43 (5.07%)	14 (4.30%)	29 (5.56%)	0.389
COPD	88 (10.38%)	35 (10.74%)	53 (10.15%)	0.843
Diabetes	149 (17.57%)	56 (17.18%)	93 (17.82%)	0.738
CKD	80 (9.43%)	16 (4.91%)	64 (11.88%)	< 0.001
RRT	103 (12.15%)	7 (2.15%)	96 (18.39%)	< 0.001
TyG index	9.02 (8.53, 9.64)	8.81 (8.35, 9.42)	9.11 (8.66, 9.80)	< 0.001

Continuous variables were expressed as mean ± standard deviation (SD) for normally distributed variables, median (IQR) for non-normally distributed continuous variables, and numbers (%) for categorical variables. The *T* test or one-way ANOVA was used to compare continuous variables, while Pearson’s *χ*^2^ test was used to compare categorical variables when examining baseline characteristics

*BMI* body mass index, *RBC* red blood cell, *WBC* white blood cell, *TG* triglyceride, *TC* total cholesterol, *ALT* alanine aminotransferase, *AST* aspartate aminotransferase, *BUN* blood–urea–nitrogen, *HDL* high density lipoprotein cholesterol, *LDL* low density lipoprotein cholesterol, *ALP* alkaline phosphatase, *CRP* C-reactive protein, *APSIII* acute physiology score III, *SIRS* systemic inflammatory response syndrome score, *SAPSII* simplified acute physiology score II, *SOFA* sequential Organ Failure Assessment score, *COPD* Chronic obstructive pulmonary disease, *RRT* renal replacement therapy, *CKD* chronic kidney disease, *TyG* triglyceride–glucose

### Primary endpoint

**Fig. 2:**
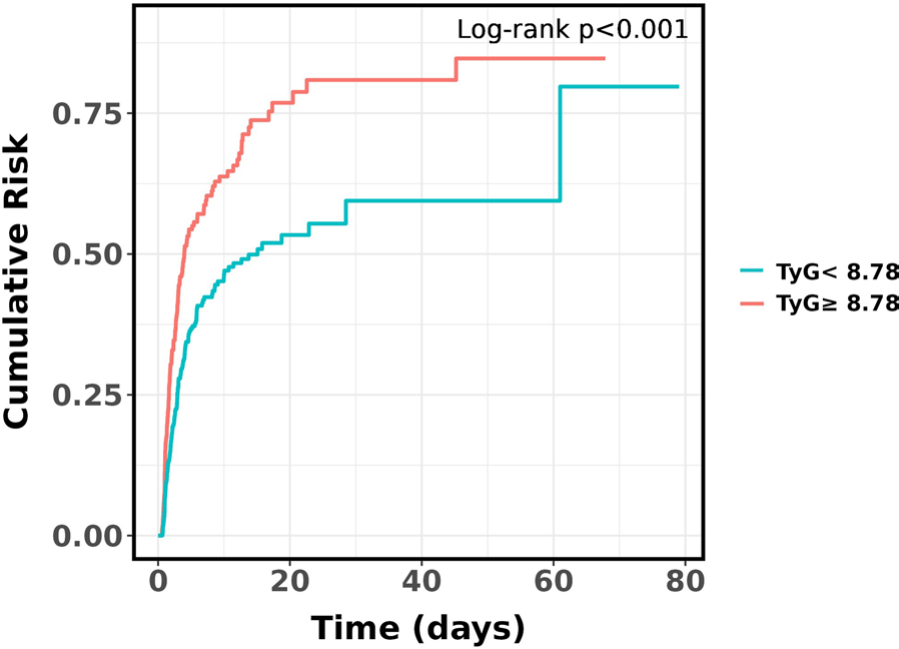
Cumulative event incidence curve for acute kidney injury incidence

Figure 2 presents the cumulative event incidence curve for the probability distribution of AKI incidence based on the TyG index. During the follow-up period, the incidence of AKI in the TyG index < 8.78 group was significantly lower than that in the TyG index ≥ 8.78 group (*P* < 0.001).

Secondary endpoints.

**Fig. 3:**
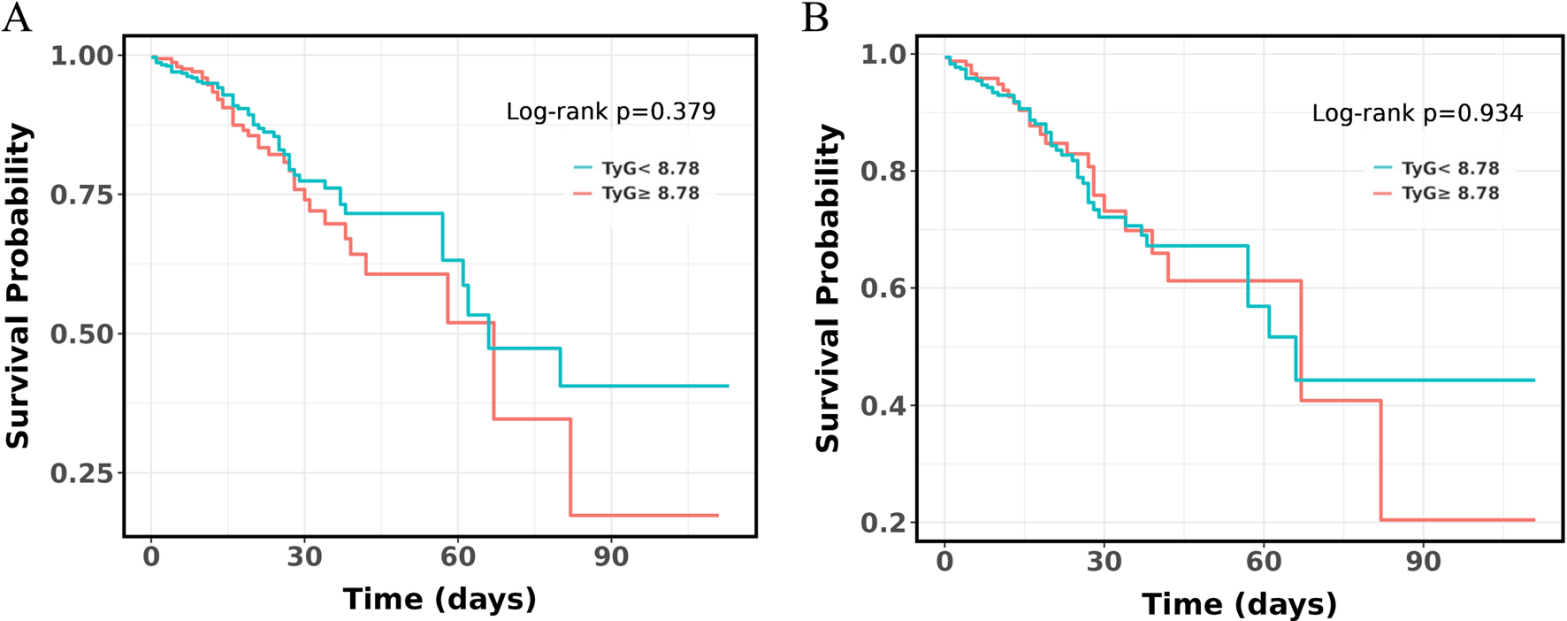
**A** Kaplan–Meier survival analysis curve for the in-hospital mortality of the whole study population; **B** Kaplan–Meier survival analysis curve for the in-hospital mortality of the acute kidney injury patients

**Fig. 4:**
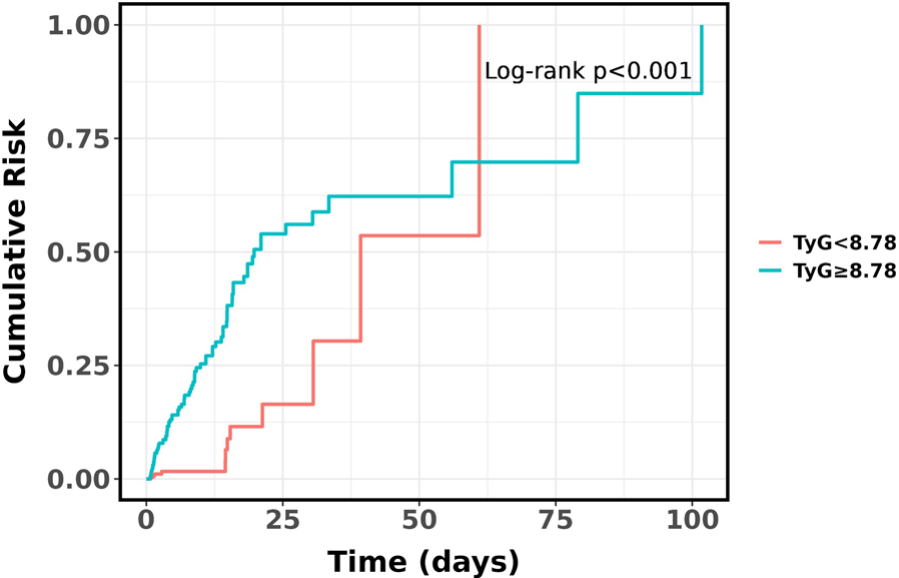
Cumulative event incidence curves for the use of renal replacement therapy of the acute kidney injury patients

Kaplan–Meier survival analyses were carried out to evaluate the effect of the TyG index on secondary endpoints in the overall study population and AKI population. No significant differences were found in the in-hospital mortality between the overall study population (*P* = 0.379, Fig. 3A) and the AKI population (P = 0.934, Fig. 3B), based on the TyG index quartiles. As shown in Fig. 4, AKI patients with TyG ≥ 8.78 are at high risk of requiring RRT (*P* < 0.001).

As shown in Table 3, multivariable Cox regression analysis showed whether TyG index was a continuous variable (HR, 1.65 [95% CI 1.10–2.48], *P* = 0.015) or a categorical variable (HR, 1.72 [95% CI 1.09–2.79], *P* = 0.028), the TyG index was independently associated with the risk of AKI in AP patients.

**Table 3:** Cox proportional hazard ratios for AKI incidence

	Crude model		Model 1		Model 2	
	HR(95% CI)	*P* value	HR(95% CI)	*P* value	HR(95% CI)	*P* value
TyG as Continuous	1.38 (1.20–1.59)	< 0.001	1.39 (1.16–1.67)	< 0.001	1.65(1.10–2.48)	0.015
TyG < 8.78	Reference		Reference		Reference	
TyG ≥ 8.78	1.59(1.18–2.71)	< 0.001	1.61 (1.11–2.98)	0.017	1.72 (1.09–2.79)	0.028

Crude model: unadjusted model;

Model 1: Adjusted for age, sex, BMI

Model 2: Adjusted for age, sex, BMI, laboratory variables (hemoglobin, RBC, WBC, neutrophils, lymphocyte, HDL, LDL, TC, TG, total bilirubin, creatinine, potassium, BUN, AST, ALT, ALP, CRP) and comorbidities(hypertension, atrial fibrillation, myocardial Infarction, COPD, CKD, diabetes)

*HR* hazard ratios, *CI* confidence interval. *BMI* body mass index, *RBC* red blood cell, *WBC* white blood cell, *HDL* high density lipoprotein cholesterol, *LDL* low density lipoprotein cholesterol, *TC* total cholesterol, *TG* triglyceride, *BUN* blood–urea–nitrogen, *AST* aspartate aminotransferase, *ALT* alanine aminotransferase, *ALP* alkaline phosphatase, *CRP* C-reactive protein, *COPD* Chronic obstructive pulmonary disease, *CKD* chronic kidney disease

**Fig. 5:**
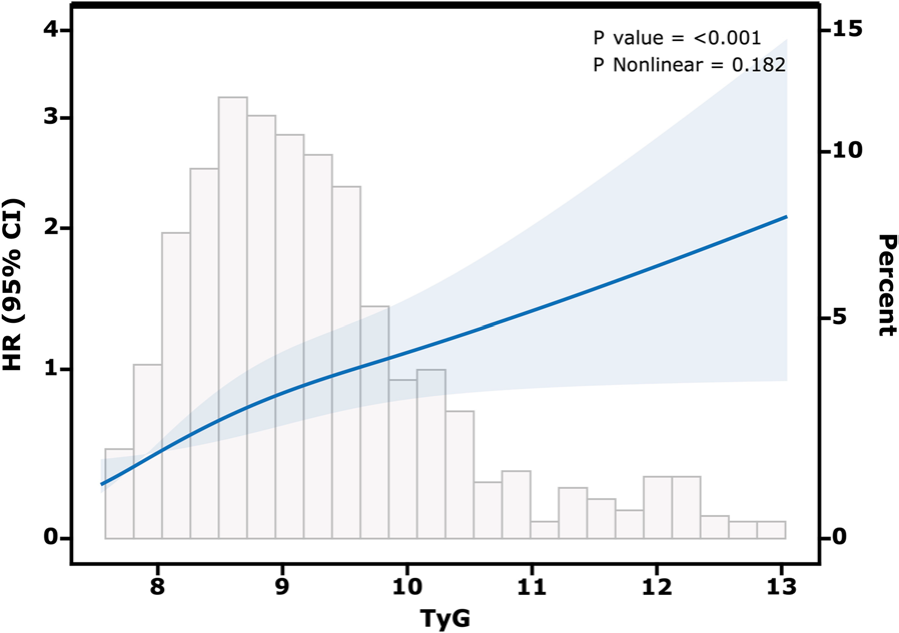
Restricted cubic spline curve of the TyG index with acute kidney injury incidence

Figure 5 shows the restricted cubic splines regression model, it suggested a linear association between the TyG index and AKI after adjusting for confounders (*P* non-linear = 0.182). When the TyG index was higher than 9.60, the risk of AKI was positively correlated with the value of the TyG index.

**Fig. 6:**
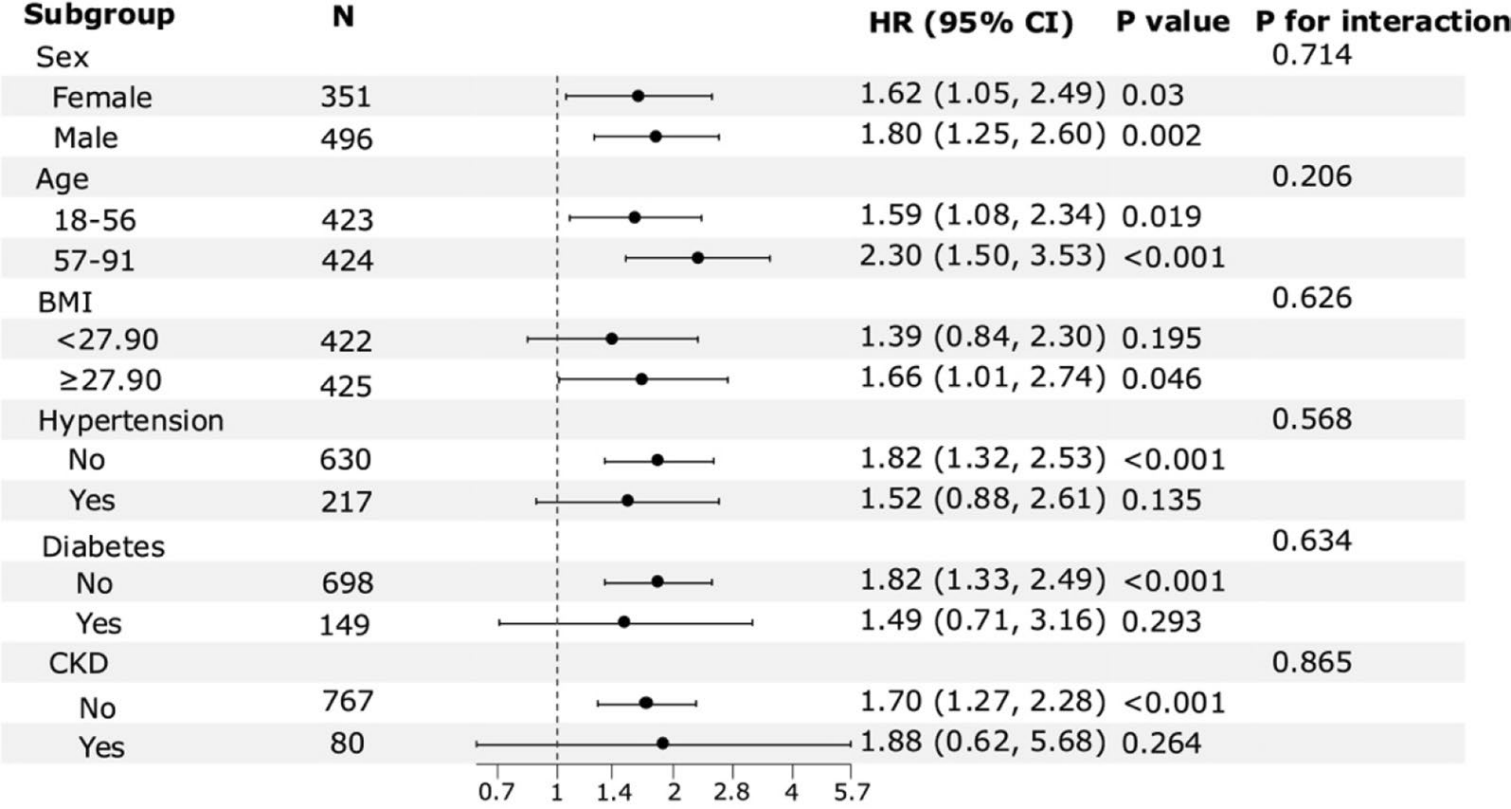
Forest plots for the primary endpoint in different subgroups. *HR* hazard ratios, *CI* confidence interval, *BMI* body mass index, *CKD* chronic kidney disease

Further stratification and interaction analyses were conducted to evaluate the relationship between TyG index and AKI based on gender, age, BMI, hypertension, diabetes, and CKD (Fig. 6). No significant interaction effect was observed between the TyG index and AKI risk (all *P* for interaction > 0.05).

## Discussion

AP is an inflammatory condition associated with a high complication rate and increased risk of death. AKI has long been considered as a common and significant complication of AP [3]. Previous study has highlighted the high prevalence of AKI in AP and its poor prognosis [16]. Early identification of AKI risk is crucial for improving the prognosis of AP. Our findings indicate that the TyG index ≥ 8.78 was associated with an increased risk of AKI in critical ill patients with AP, and this association remained statistically significant even after adjusting for potential confounders. RCS model suggested that the risk of AKI in patients with AP was linearly correlated with the TyG index.

The prevalence of AKI in AP has not been well established. In a comprehensive, retrospective, observational study, Devani et al. reported an overall AKI prevalence of 7.9% among 3,466,493 patients hospitalized with AP. The mortality was significantly higher in the AKI group(8.8 vs. 0.7%; *p* < 0.01). In the matched patients, AKI significantly increased the risk of death (Propensity-matched OR: 3.20, 95% CI 2.90–3.51, *p* < 0.001) [16]. However, in a study focused on ICU patients, a significant proportion (72.1%) of AP patients develop AKI in the ICU, which is notably higher than the hospitalized AP patients [17]. The incidence of AKI in patients with severe AP is 15–70% [6, 18]. In this study, the incidence of AKI was 61.56%, and the in-hospital mortality was 11.69%. Given the severity of the disease and the high incidence of AKI in the ICU, we consider these results to be acceptable. Therefore, there is an urgent need to explore new biomarkers to identify AP patients at a high risk of developing AKI in the ICU to improve their prognosis.

The TyG index, originally used as a valid, cost-effective, and reproducible indicator of IR in diabetes, has been recognized for its utility in various diseases, including metabolic disorders, cardiovascular diseases, atherosclerotic diseases, and even Corona Virus Disease 2019 [19–23]. There remains a scarcity of studies exploring the association between the TyG index and AP. A retrospective study found that high TyG index was associated with severe AP and its complications [14]. Some researchers have identified the TyG index as an effective biomarker for identifying severe AP [13]. As the TyG index threshold value, our study's RCS model suggest that higher TyG index above 9.60 may correlate with the risk of AKI in patients with AP. In terms of kidney disease, the TyG index can be used to predict the incidence and progression of a variety of kidney diseases, including diabetic nephropathy and aging nephropathy [24, 25]. Shi et al. found in a retrospective cohort study that a high TyG index was an independent risk factor for AKI and adverse renal outcomes in patients undergoing coronary revascularization. They suggested that the TyG index is a simple and efficient biomarker for AKI risk stratification [26]. Similarly, Yang et al. found a significant linear relationship between the TyG index levels and the risk of AKI in patients with critical heart failure [22]. Those evidences collectively highlight the TyG index is a reliable and independent predictor of adverse renal outcomes, potential may be useful for risk stratification of AKI in AP patients. Our findings suggest that, the risk of AKI in critical ill AP patients with the TyG index ≥ 8.78 was 1.78 times higher than that in patients with the TyG index < 8.78. In addition, the risk of AKI increased 1.65-fold for each unit increase in the TyG index. Therefore, in clinical practice, the changes of TyG index should be evaluated regularly, and the occurrence of AKI should be prevented in patients with the TyG index ≥ 8.78 or the TyG index continuously increased.

The premature activation of pancreatic enzymes in the acinar cells leads to pancreatic autodigestion and release of enzymes and proteases, triggering a serious of events that are involved in the pathogenesis of AKI in AP patients [27]. Hypotension and hypovolemia may be the initial factors of AKI in the early stage of AP. Toxic agents in the pancreatic exudate, including trypsin, chymotrypsin, bradykinin, histamine, and prostaglandin E, as well as endotoxins and bacteria, may subsequently contribute to AKI [2, 28, 29]. Previous researches have identified certain clinical biomarkers associated with AKI in AP, including angiopoietin-2, procalcitonin, neutrophil gelatinase-associated lipocalin, monocyte chemoattractant protein, uromodulin [9, 30–32]. In addition, the TyG index can serve as a simple predictor for assessing the degree of IR, and is strongly associated with renal injury [33].Through multivariable regression analysis and subgroup analysis, we found that the TyG index was independently associated with the risk of AKI in critical ill patients with AP. The TyG index is conducive to optimizing the risk stratification of AKI in AP patients. Regular evaluation of the TyG index is helpful for early clinical intervention and improvement of prognosis.

This study has some limitations. First, due to the retrospective design of this study, we could not definitively establish causality. Besides, the study population was mainly comprised of individuals from the United States, indicating that the relationship between TyG index and risk of AKI in other races, such as the Asian population, requires further validation. Second, on our investigation of the relationship between between the initial TyG index after admission and the risk of AKI in patients with AP, dynamic monitoring of the TyG index is needed in further research, as some patients were receiving anti-diabetic and lipid-lowering therapies, which inevitably affect the stability of the index. These omissions may contribute to potential bias in the study’s outcomes.

## Conclusion

High TyG index is an independent risk factor for AKI in critical ill patients with AP. Assessing the TyG index may be beneficial for early stratification and interventions to improve prognosis.

## Data Availability

The data that support the findings of this study are available from the corresponding author upon reasonable request.

## Abbreviations

AP: Acute pancreatitis
AKI: Acute kidney injury
ICU: Intensive care unit
IR: Insulin resistance
TyG: Triglyceride glucose
MIMIC IV: Medical Information Mart for Intensive Care IV
TG: Triglyceride
BMI: Body mass index
RBC: Red blood cell
WBC: White blood cell
HDL: High density lipoprotein cholesterol
LDL: Low density lipoprotein cholesterol
TC: Total cholesterol
BUN: Blood–urea–nitrogen
AST: Aspartate aminotransferase
ALT: Alanine aminotransferase
ALP: Alkaline phosphatase
CRP: C-reactive protein
SAPSII: Simplified acute physiology score II
SIRS: Systemic inflammatory response syndrome
APSIII: Acute physiology scores III
SOFA: Sequential Organ Failure Assessment
COPD: Chronic obstructive pulmonary disease
CKD: Chronic kidney disease
RRT: Renal replacement therapy
HR: Hazard ratio
CI: Confidence interval
RCS: Restricted cubic spline
